# Lower CALLY index levels indicate higher poor functional outcome risk in acute ischemic stroke patients treated with endovascular thrombectomy

**DOI:** 10.3389/fnagi.2025.1587861

**Published:** 2025-04-25

**Authors:** Yunnan Lu, Xiaohua Zhu, Yaojia Xu, Yongxin Li, Qingyong Dai, Xia Chang

**Affiliations:** ^1^Department of Neurology, Xishan People’s Hospital of Wuxi City, Wuxi, China; ^2^Department of Neurology, The Affiliated Zhangjiagang Hospital of Soochow University, Suzhou, China

**Keywords:** C-reactive protein-albumin-lymphocyte, stroke, endovascular, thrombectomy, inflammation, nutrition

## Abstract

**Background:**

The imbalance in the nutrition-immunity-inflammation status is linked to the prognosis of various diseases. This study sought to evaluate the correlation between the C-reactive protein-albumin-lymphocyte (CALLY) index and the outcomes of acute ischemic stroke (AIS) managed with endovascular thrombectomy (EVT).

**Methods:**

This study retrospectively enrolled 473 AIS patients who underwent EVT from a multicenter investigation. Poor functional outcome was defined as a modified Rankin scale score exceeding 2 points at 90 days after EVT. The cutoff value for the CALLY index was determined using the receiver operating characteristic curve. Multivariable logistic regression models were utilized to explore the association between the CALLY index and poor functional outcome and restricted cubic splines was used to illustrate the relationship between the CALLY index and the risk of poor functional outcome after EVT.

**Results:**

Poor functional outcomes occurred in 214 (45.2%) patients at 90 days after EVT. The cutoff for the CALLY index was 10^ (−0.635). Multivariate logistic regression revealed that the CALLY index was significantly associated with poor functional outcome (odds ratio [OR]: 0.80, 95% confidence interval [CI]: 0.70–0.91, *p* < 0.001; high versus low OR: 0.64, 95% CI: 0.41–1.00, *p* = 0.048). The restricted cubic spline analysis indicated an inverse association between the CALLY index and the risk of poor functional outcome (*P* for nonlinearity = 0.373).

**Conclusion:**

Our study identified that a lower CALLY index is an independent predictor of poor functional outcome after EVT. The CALLY index could emerge as a practical, cost-effective, and promising predictive biomarker for adverse outcomes in AIS patients undergoing EVT treatment.

## Introduction

Acute ischemic stroke (AIS) caused by large vessel occlusion (LVO) is a devastating disease that is associated with high morbidity and mortality rates (Saini et al., 2021). Endovascular thrombectomy (EVT) is an effective treatment for early recanalization of occluded large cerebral arteries in AIS patients, aiming to preserve the ischemic penumbra (Goyal et al., 2016; Lin et al., 2019). However, over 50% of patients still experience unfavorable outcomes, with nearly one in six patients die within 90 days after EVT (Saver et al., 2016). Despite successful vessel recanalization, reperfusion injury can lead to early complications, such as neurological deterioration, potentially exacerbating long-term functional recovery (Seners and Baron, 2018; Wang and Xiong, 2023). Hence, early identification of patients at higher risk for unfavorable outcome is crucial to target timely interventions, including anti-inflammatory therapy or intensive nutritional support.

The C-reactive protein-albumin-lymphocyte (CALLY) index, derived from the combined measurement of C-reactive protein (CRP), serum albumin, and lymphocyte counts, is a novel and comprehensive indicator for assessing both nutritional and inflammatory status (Müller et al., 2021). Inflammatory and thrombotic responses to acute cerebral ischemia and reperfusion injury are significant factors in the occurrence of adverse outcomes and subsequent progression of long-term outcomes (Liu et al., 2021; De Meyer et al., 2022). Previous studies found that neutrophils and monocytes have a deleterious effect, while lymphocytes play a protective role in the prognosis of AIS (Carmona-Mora et al., 2023; Li et al., 2023). Consequently, neutrophil to lymphocyte ratio (NLR) has been reported as an independent predictor for the occurrence of intracerebral hemorrhage and poor outcome after stroke (Pikija et al., 2018). CRP, as an inflammatory biomarker, reflects inflammation triggered by cerebral ischemia and post-stroke infection, and is associated with functional outcomes after EVT (Seo et al., 2012). Notably, nutritional status significantly impacts the prognosis of AIS patients (Zielińska-Nowak et al., 2021). Albumin can mediate systemic inflammation and exerting antioxidant activity, thereby protecting brain tissues and influencing the chance of recovery after stroke (Gao et al., 2021; Luo et al., 2022). The CALLY index, by integrating both inflammatory and nutritional markers, systematically evaluates patient health and outperforms traditional indexes in predicting clinical outcomes across a range of diseases. Previous studies have shown its association with poor outcomes of patients with gastric cancer treated with gastrectomy (Fukushima et al., 2024). Additionally, the CALLY index has been validated for predicting the prognosis of patients with various types of tumors (Jia et al., 2025) and cardiovascular diseases such as ST-segment elevation myocardial infarction (Ji et al., 2024). Given its high accessibility and cost-effectiveness, the CALLY index may provide valuable information for prognostication and treatment planning in AIS patients.

However, the association between the CALLY index and clinical outcomes after EVT in AIS patients remained unexplored. Therefore, this study aimed to investigate the predictive capability of the CALLY index for poor functional outcomes in AIS patients treated with EVT in a multicenter study, with the hope of aiding clinical screening for high-risk patients.

## Methods

Data that support the findings of this study were available from the corresponding authors upon reasonable request.

### Study population

This multicenter retrospective study was conducted at the Xishan People’s Hospital of Wuxi City and The Affiliated Zhangjiagang Hospital of Soochow University from June 2021 to September 2024. We included patients according to the following criteria: (1) age ≥18 years; (2) AIS patients treated with EVT within 24 h of symptom onset; (3) LVO in the anterior circulation (internal carotid artery, M1 or M2 segment of the middle cerebral artery); (4) pre-stroke modified Rankin Scale (mRS) score < 2; (5) National Institute of Health Stroke Scale (NIHSS) score ≥ 6; and (6) ASPECTS score ≥ 5. We exclude patients with (1) incomplete baseline CRP, albumin, and lymphocyte count; (2) acute or chronic infectious diseases; (3) autoimmune diseases; and (4) loss to follow-up. We included 473 patients in the final analysis. This study was approved by the ethics committee of each participating center and conducted in accordance with the ethical principles outlined in the 1964 Declaration of Helsinki and its subsequent amendments. Given the retrospective design of the study, the requirement for written informed consent was waived.

### Data collection

We collected clinical variables in this study including: demographic data, physical examination, comorbidities, laboratory values, angiographic outcomes, stroke severity measured by the National Institutes of Health Stroke Scale (NIHSS) (Brott et al., 1989), and stroke subtypes as assessed by the Trial of ORG 10172 in Acute Stroke Treatment (TOAST) classification (Adams et al., 1993). Brain computed tomography (CT) and magnetic resonance imaging (MRI) were performed routinely upon admission and repeated within 24 h after EVT or in the event of neurological deterioration. Radiological images were independently reviewed by two experienced neurologists, who were blinded to the study details. The degree of brain ischemia was assessed by the Alberta Stroke Program Early CT Score (ASPECTS) (Barber et al., 2000). Successful recanalization was defined as a modified Thrombolysis in Cerebral Ischemia (mTICI) score of 2b / 3 after EVT.

### Laboratory testing

Prior to EVT, patients were admitted to the emergency department to get the blood sampling from the antecubital vein for the routine blood examination and biochemical analysis immediately. The CALLY index was calculated using the following formula: albumin × lymphocyte count / (CRP × 10) (Müller et al., 2021). NLR = neutrophil / lymphocyte count, platelet to lymphocyte ratio (PLR) = platelet / lymphocyte count, systemic immunoinflammatory index (SII) = neutrocyte × platelet / lymphocyte count.

### Treatment

Neuro-interventionists with enough experience would perform EVT procedures, employing stent retrievers, aspiration thrombectomy, or a combination of these methods. For patients eligible for intravenous thrombolysis (IVT), urokinase, alteplase, or tenecteplase were administered within their respective therapeutic time frames after onset. Rescue therapies would be contemplated in instances where target vessel recanalization was unsuccessful, involving intracranial angioplasty, intra-arterial thrombolysis, tirofiban infusion, or stent placement.

### Clinical outcome

At 90 days after EVT, functional outcomes were evaluated utilizing the mRS score, a prevalent clinical metric for assessing stroke patients’ disability and dependency levels. The evaluations were independently performed by two trained neurologists, who were unaware of the study’s design, via standardized interviews. Poor functional outcome was defined as an mRS score exceeding 2 at the 90-day mark. Early neurological deterioration (END) was characterized by an increase of ≥ 4 points in the NIHSS score from baseline to the assessment at 48 h after EVT (Kim et al., 2019).

### Statistical analysis

Data were presented as mean ± standard deviation (SD), median with interquartile ranges (IQR) or count with percentages (n [%]). Baseline characteristics were evaluated using Fisher’s exact test or chi-square tests. For continuous variables exhibiting normal distributions, Student’s *t*-tests were employed, whereas the Mann–Whitney test was utilized for those with non-normal distributions. We performed the Little’s MCAR test to assess the pattern of missing data. Variables with missing values (height, weight, uric acid, creatinine, and glucose) were imputed with five multiple imputations via chained equations and the variables were missing at random (*p* = 0.854).

The receiver operating characteristic (ROC) curve was employed to assess the CALLY index’s discriminative ability in predicting poor functional outcomes. Delong’s test was employed to assess the accuracy of the CALLY index in comparison with NLR, SII, and PLR. The cut-off point for the CALLY index was determined based on the point with the highest Youden index. Given the skewed distribution of the CALLY index, a logarithmic transformation was applied to achieve a normal distribution (Supplementary Figure S1). We then used multivariable logistic regression models to evaluate the association between CALLY index and clinical outcomes after EVT. Model 1 was the unadjusted model. Model 2 was adjusted for medical history: smoke, hypertension, diabetes mellitus, hyperlipidemia, coronary heart disease, atrial fibrillation and cancer. Model 3 was adjusted for variables with *p* < 0.1 in the univariable analysis after the back-ward selection. Logistic regression model results were reported as odds ratio (OR) with corresponding 95% confidence interval (CI).

To illustrate the association between the CALLY index and poor functional outcome, a restricted cubic spline analysis was conducted with three knots positioned at the 10th, 50th, and 90th percentiles based on the distribution of the CALLY index (Supplementary Figure S2), while accounting for the variables included in model 3. Furthermore, we evaluated the enhancement in discriminative capability by integrating the CALLY index into model 2 and model 3, utilizing the net reclassification improvement (NRI) and integrated discrimination improvement (IDI) metrics. In sensitivity analyses, subgroup analyses were performed, stratified by age, sex, hypertension, diabetes mellitus, atrial fibrillation, TOAST classification, IVT, and NIHSS score to confirm the robustness of the results. To investigate the changes in post-EVT biomarkers, we categorized patients into those with increased and decreased CALLY index based on available data.

All statistical analyses were conducted with R statistical software version 4.2.2 (R Foundation, Vienna, Austria), and a two-sided *p* value < 0.05 was considered to be statistically significant.

## Results

Of the 652 enrolled patients, we excluded 172 patients due to incomplete data and an additional 7 patients with infectious or autoimmune diseases (Supplementary Figure S3). We included 473 patients in the final analysis, the median age was 71 years (IQR: 64–79 years), NIHSS was 13 (IQR: 10–17 points). The missing pattern was completely at random (*p* = 0.608). Baseline characteristics were summarized in Table 1. 302 (63.8%) patients were male and 179 (37.8%) received IVT. Large artery atherosclerosis affected 218 (46.1%) patients. 442 (93.4%) patients achieved successful recanalization. Patients with a poor functional outcome were characterized by being older, having a higher prevalence of diabetes mellitus and atrial fibrillation, longer puncture-to-reperfusion times, higher NIHSS scores, elevated fasting blood glucose levels, and increased inflammatory markers such as CRP, WBC, and neutrophils counts, a lower proportion of successful recanalization and END, and lower ASPECTS scores. Most characteristics were comparable across centers, with the exception of the proportion of IVT, onset to puncture and puncture to reperfusion time (*p* < 0.05; Supplementary Table S1).

**Table 1:** Characteristics of the study population according to the functional outcome after EVT.

Variable	Total (*n* = 473)	Poor outcome (*n* = 214)	Good outcome (*n* = 259)	*p* value
Age (years)	71.0 [64.0, 79.0]	75.0 [69.0, 82.0]	68.0 [59.5, 76.0]	<0.001
Gender/male, *n* (%)	302 (63.8)	121 (56.5)	181 (69.9)	0.004
Physical examination				
Height (cm)	165.0 [160.0, 170.0]	165.0 [158.0, 170.0]	167.0 [160.0, 170.0]	0.057
Weight (kg)	65.0 [60.0, 75.0]	65.0 [55.0, 75.0]	68.0 [60.0, 75.0]	0.094
SBP (mmHg)	139.2 (22.2)	140.9 (24.0)	137.7 (20.5)	0.125
DBP (mmHg)	83.9 (13.4)	83.5 (14.2)	84.2 (12.7)	0.581
Comorbidities, *n* (%)				
Smoking	183 (38.7)	63 (29.4)	120 (46.3)	<0.001
Drinking	109 (23.0)	38 (17.8)	71 (27.4)	0.018
Hypertension	338 (71.5)	160 (74.8)	178 (68.7)	0.178
Diabetes mellitus	131 (27.7)	76 (35.5)	55 (21.2)	0.001
Hyperlipidemia	45 (9.5)	19 (8.9)	26 (10.0)	0.787
Coronary artery disease	72 (15.2)	39 (18.2)	33 (12.7)	0.128
Atrial fibrillation	129 (27.3)	76 (35.5)	53 (20.5)	<0.001
Cancer	16 (3.4)	9 (4.2)	7 (2.7)	0.519
Laboratory values (median [IQR])				
UA (μmol/L)	311.6 [247.4, 386.2]	316.9 [253.5, 393.4]	304.0 [240.8, 378.4]	0.189
Serum creatinine (μmol/L)	72.0 [59.9, 88.5]	75.7 [60.2, 93.9]	70.9 [59.0, 85.6]	0.084
CRP (mg/L)	8.2 [3.0, 27.0]	11.0 [4.9, 43.5]	7.0 [2.0, 18.2]	<0.001
Albumin (g/L)	38.9 [35.5, 41.9]	38.5 [34.3, 41.5]	39.4 [36.2, 42.5]	0.017
WBC count (×10^9^/L)	9.3 [7.2, 12.2]	9.9 [7.6, 13.1]	9.1 [7.0, 11.6]	0.003
Neutrophil count (×10^9^/L)	7.8 [5.6, 10.4]	8.2 [5.9, 11.2]	7.3 [5.0, 9.8]	0.002
Lymphocyte count (×10^9^/L)	1.0 [0.7, 1.5]	1.0 [0.7, 1.5]	1.1 [0.7, 1.5]	0.416
Glu (mmol/L)	6.7 [5.7, 8.6]	6.8 [5.9, 9.0]	6.6 [5.4, 8.2]	0.002
PLT count (×10^9^/L)	172.0 [137.0, 216.0]	177.0 [138.0, 219.8]	170.0 [134.5, 213.0]	0.435
TOAST, *n* (%)				0.003
CES	211 (44.6)	112 (52.3)	99 (38.2)	
LAA	218 (46.1)	89 (41.6)	129 (49.8)	
Other	44 (9.3)	13 (6.1)	31 (12.0)	
IVT, *n* (%)	179 (37.8)	76 (35.5)	103 (39.8)	0.393
Angiographic outcomes				
Number of attempts (*n*)	1.0 [1.0, 2.0]	2.0 [1.0, 3.0]	1.0 [1.0, 2.0]	<0.001
mTICI 2b/3, *n* (%)	442 (93.4)	190 (88.8)	252 (97.3)	<0.001
OTP (min)	294.0 [180.0, 495.0]	295.0 [190.0, 473.2]	290.0 [180.0, 527.5]	0.713
PTR (min)	60.0 [43.0, 93.0]	70.0 [50.2, 102.0]	55.0 [40.0, 77.0]	<0.001
ASITN/SIR 2–3, *n* (%)	64 (13.5)	24 (11.2)	40 (15.4)	0.229
Clinical scores (points)				
Baseline NIHSS	13.0 [10.0, 17.0]	14.0 [12.0, 19.0]	12.0 [8.0, 16.0]	<0.001
Baseline mRS	0.0 [0.0, 0.0]	0.0 [0.0, 0.0]	0.0 [0.0, 0.0]	<0.001
Baseline ASPECTS	9.0 [8.0, 9.0]	8.0 [8.0, 9.0]	9.0 [8.0, 9.0]	<0.001
Occlusion site, *n* (%)				0.209
ICA	110 (23.3)	52 (24.3)	58 (22.4)	
MCA-M1	243 (51.4)	100 (46.7)	143 (55.2)	
MCA-M2	39 (8.2)	18 (8.4)	21 (8.1)	
T occlusion	81 (17.1)	44 (20.6)	37 (14.3)	
END, *n* (%)	88 (18.6)	57 (26.6)	31 (12.0)	<0.001
Inflammation indexes				
NLR	7.2 [4.1, 12.6]	8.0 [4.7, 14.3]	6.4 [3.9, 11.4]	0.006
SII	1238.6 [675.8, 2159.6]	1325.6 [729.0, 2518.7]	1160.4 [621.2, 1950.1]	0.005
PLR	157.4 [106.0, 236.7]	167.6 [107.6, 246.8]	154.6 [103.5, 230.6]	0.213
CALLY	216 (45.7)	76 (35.5)	140 (54.1)	<0.001

ASITN/SIR, the American Society of Interventional and Therapeutic Neuroradiology/Society of Interventional Radiology; ASPECTS, the Alberta Stroke Program Early Computed Tomography Score; CALLY, C-reactive protein-albumin-lymphocyte; CES, cardioembolism; CRP, C-reactive protein; DBP, diastolic blood pressure; END, early neurological deterioration; EVT, endovascular thrombectomy; Glu, fasting blood glucose; ICA, internal carotid artery; ICH, intracranial hemorrhage; IVT, intravenous thrombolysis; LAA, large artery atherosclerosis; MCA, middle cerebral artery; mRS, modified Rankin Scale Score; mTICI, modified Thrombolysis in Cerebral Infarction Score; NIHSS, National Institute of Health Stroke Scale; NLR, neutrophil/lymphocyte; OTP, from onset to puncture; PLR, platelet/lymphocyte; PTR, from puncture to recanalization; SBP, systolic blood pressure; SII, systemic immunoinflammatory index; TOAST, the trial of ORG 10172 in Acute Stroke Treatment classification; UA, uric acid; WBC, white blood cell.

**Figure 1:**
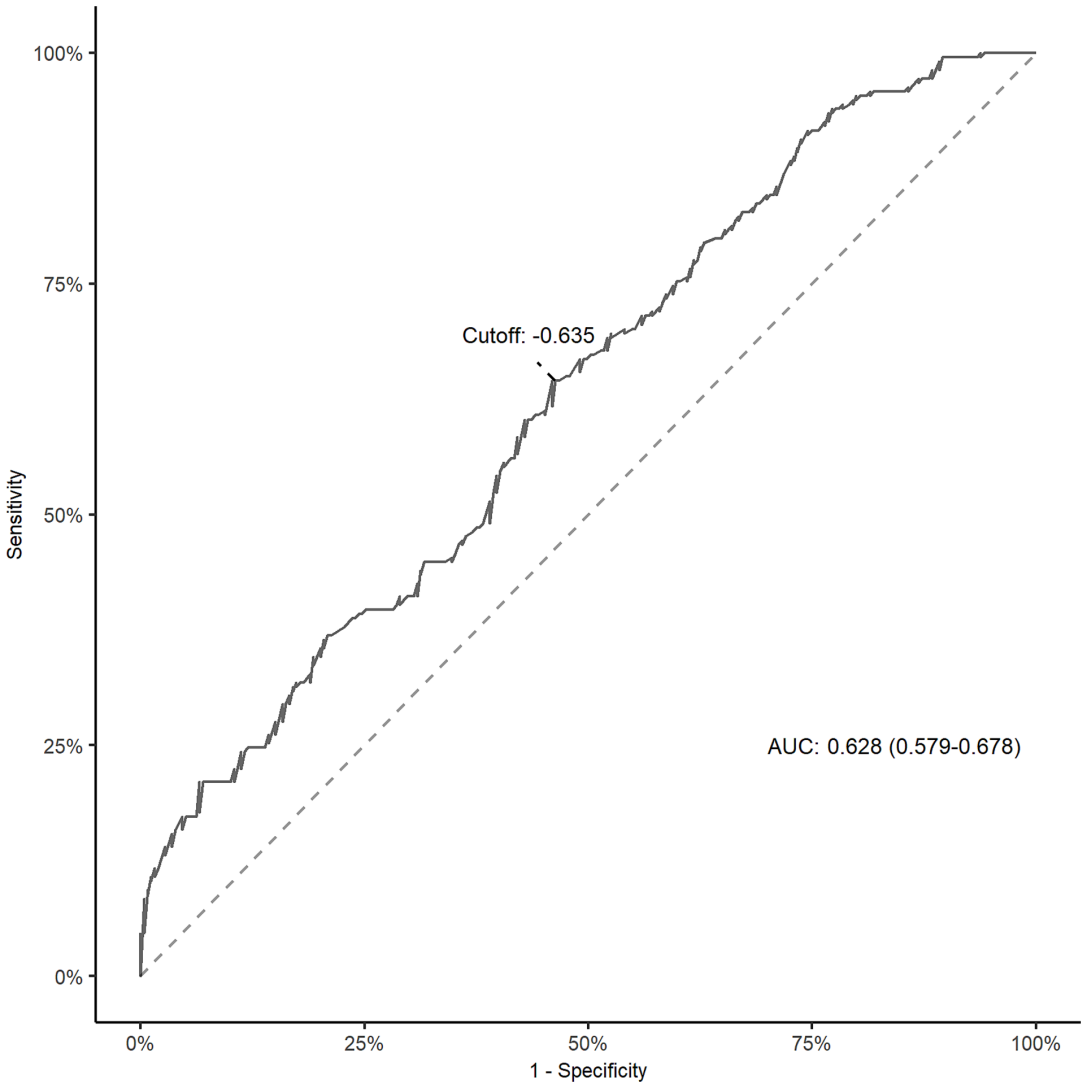
ROC curve for determining the cut-off point of the CALLY index in AIS patients treated with EVT. AIS, acute ischemic stroke; AUC, area under the curve; CALLY, C-reactive protein-albumin-lymphocyte; EVT, endovascular thrombectomy; ROC, receiver operating characteristic curve.

**Figure 2:**
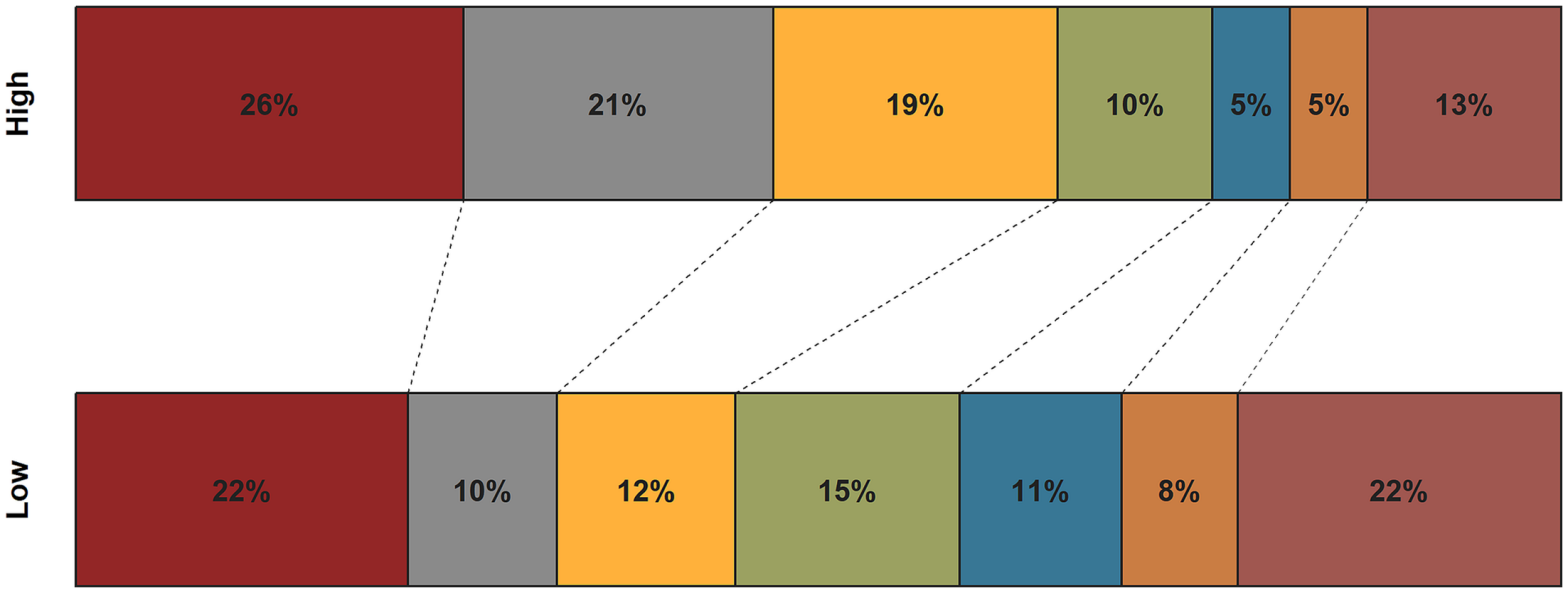
The distribution of the 90-day mRS score based on the CALLY index threshold grouping. CALLY, C-reactive protein-albumin-lymphocyte; mRS, modified Rankin Scale Score.

**Figure 3:**
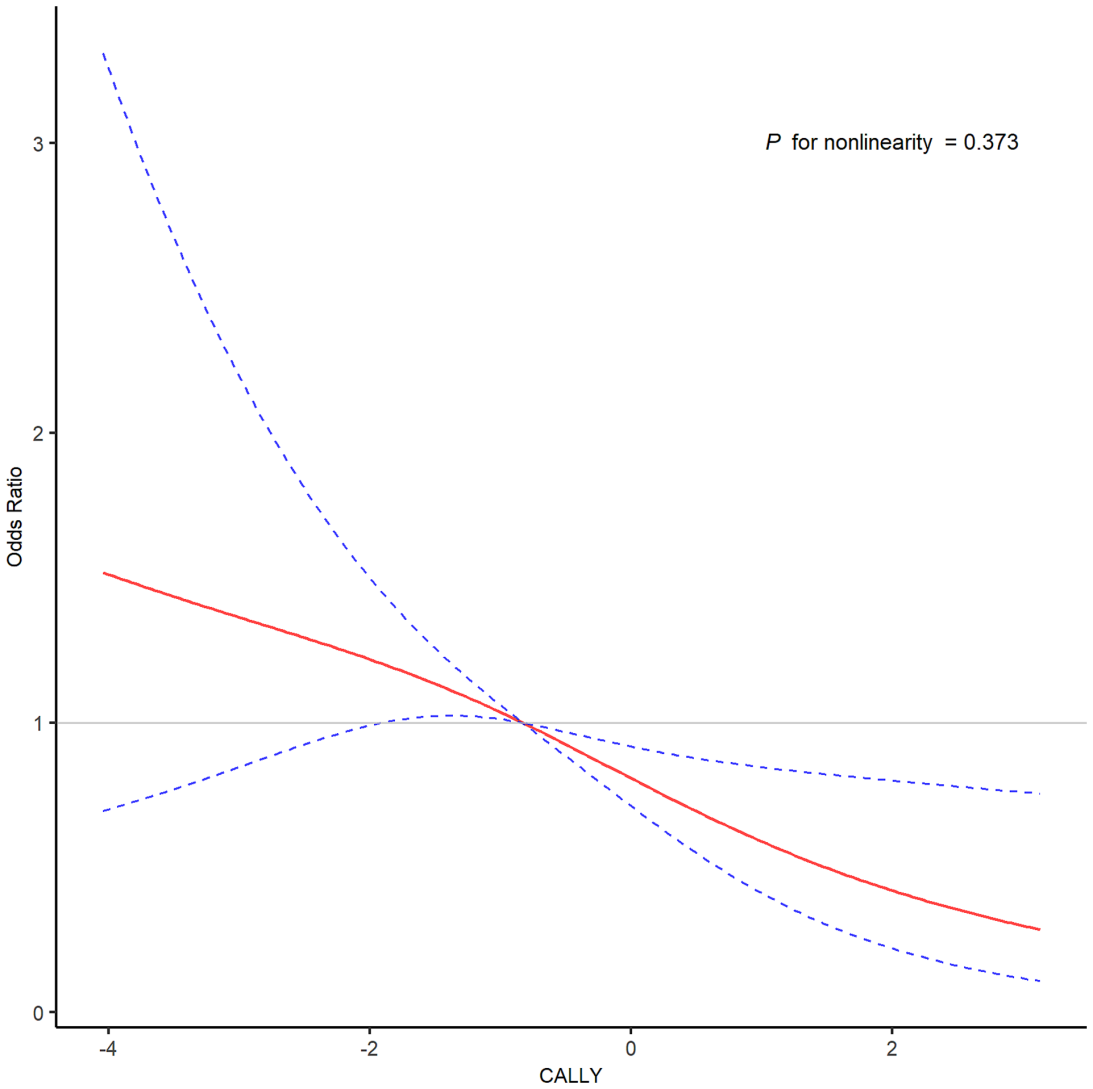
Analysis of restricted cubic spline regression to examine the association between the CALLY index and poor functional outcome after EVT. CALLY, C-reactive protein-albumin-lymphocyte; EVT, endovascular thrombectomy.

As shown in Figure 1, the ROC curve showed that the area under the curve (AUC) for the CALLY index was 0.628 (95% CI, 0.579–0.678). The Delong’s test showed that the CALLY index was better than NLR (AUC: 0.574, *p* = 0.072), SII (AUC: 0.576, *p* = 0.099) and PLR (AUC: 0.533, *p* = 0.002). The optimal cut-off value for the CALLY index was 10 ^ (−0.635). After dividing patients into low and high groups based on the cutoff value, patients with a lower CALLY index had a poorer functional outcome when compared to those in the higher group (Figure 2). The low CALLY group comprised 257 patients, while the high CALLY group included 216 patients. In the low CALLY group, 138 patients (53.7%) experienced poor outcomes, compared to 76 patients (35.2%) in the high CALLY group. Besides, patients in the low CALLY index group were older, had a longer onset to puncture time and puncture to reperfusion time, a higher creatinine, NIHSS score, and inflammatory markers (Supplementary Table S2). The restricted cubic spline curve revealed a linear relationship between the CALLY index and the risk of poor functional outcome (*P* for nonlinearity = 0.373; Figure 3).

**Figure 4:**
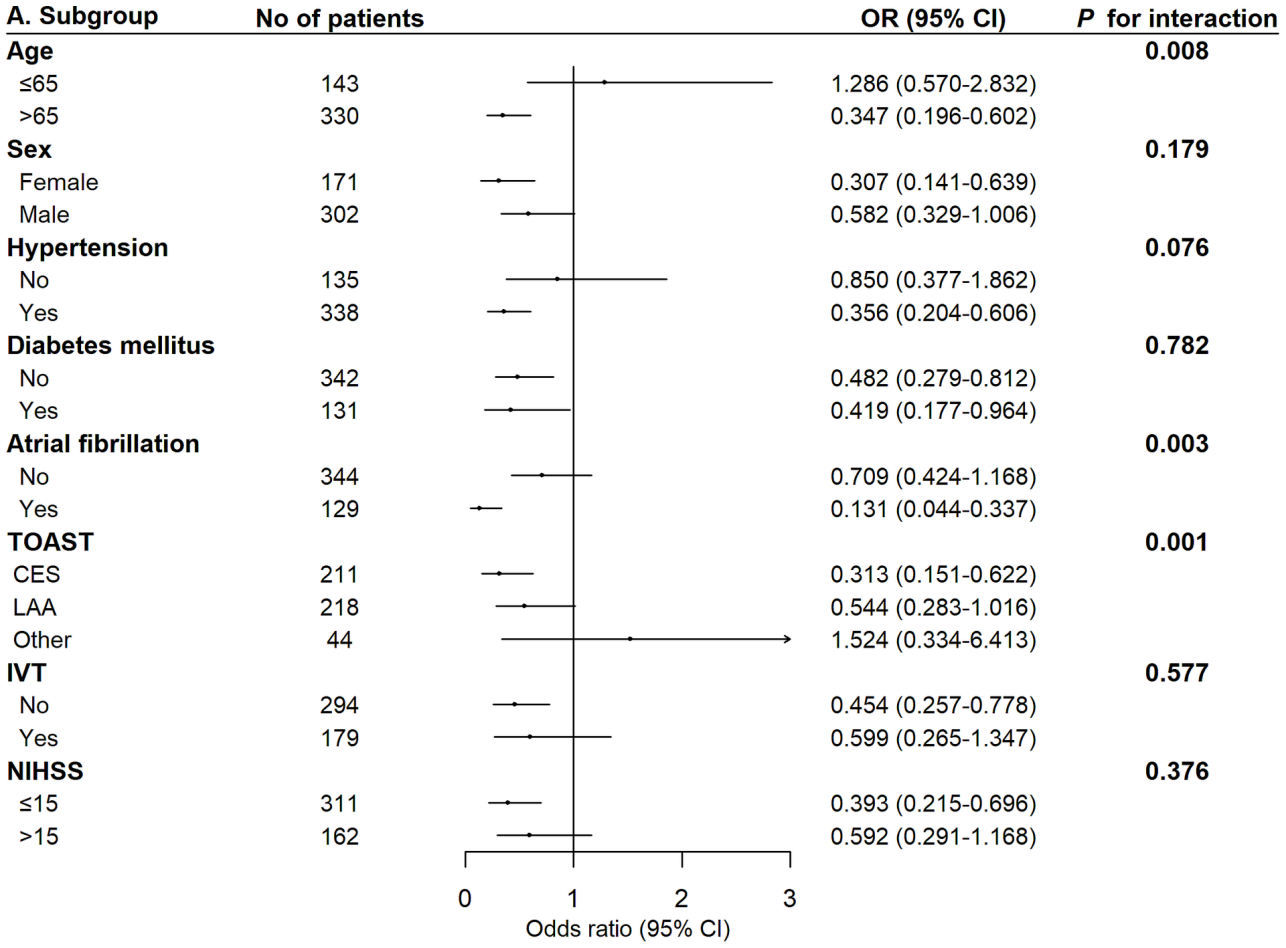
Association between the CALLY index and poor functional outcome after EVT in different stratification. CALLY, C-reactive protein-albumin-lymphocyte; CI, confidence interval; EVT, endovascular thrombectomy; IVT, intravenous thrombolysis; NIHSS, National Institute of Health Stroke Scale; OR, odds ratio; TOAST, TOAST, the trial of ORG 10172 in Acute Stroke Treatment classification.

In the univariate logistic regression analysis showed that the CALLY index was associated with poor functional outcome (OR: 0.73, 95% CI: 0.65–0.82, *p* < 0.001; high versus low OR: 0.47, 95% CI, 0.32–0.68, *p* < 0.001) and END (OR: 0.82, 95% CI: 0.71–0.93, *p* = 0.003; high versus low OR: 0.62, 95% CI, 0.38–1.00, *p* = 0.053) in the unadjusted model, in model 2 (poor functional outcome OR: 0.76, 95% CI: 0.67–0.85, *p* < 0.001; high versus low OR: 0.52, 95% CI, 0.35–0.77, *p* < 0.001; END OR: 0.82, 95% CI: 0.71–0.94, *p* = 0.006; high versus low OR: 0.64, 95% CI, 0.39–1.03, *p* = 0.070), and model 3 (poor functional outcome OR: 0.80, 95% CI: 0.70–0.91, *p* < 0.001; high versus low OR: 0.64, 95% CI, 0.41–1.00, *p* = 0.048; END OR: 0.82, 95% CI: 0.71–0.95, *p* = 0.009; high versus low OR: 0.65, 95% CI, 0.39–1.07, *p* = 0.096; Table 2). The reclassification analysis indicated a modest improvement of predictive ability by adding the CALLY index into the models (NRI: 0.214, 95% CI: 0.094–0.379, *p* < 0.001; IDI: 0.041, 95% CI: 0.023–0.058, *p* < 0.001; Table 3) Meanwhile, other inflammatory markers such as SII and NLR were associated with poor functional outcome (*p* < 0.05; Supplementary Table S3; Supplementary Figure S4). Subgroup analysis revealed an interaction between age, atrial fibrillation, and TOAST classification with the CALLY index in relation to poor functional outcome after EVT. The impact of the CALLY index may be more pronounced in the elderly population and cardio-embolic stroke (Figure 4). Additionally, among patients with available post-EVT biomarkers (*n* = 257), an increased post-EVT CALLY index was associated with poor functional outcome (model 3: OR: 0.38, 95% CI, 0.20–0.70, *p* = 0.002; Supplementary Table S4).

**Table 2:** The relationship between the CALLY index and outcomes after EVT.

	Model 1		Model 2		Model 3	
Outcome	OR (95% CI)	*P* value	OR (95% CI)	*P* value	OR (95% CI)	*P* value
**Poor outcome**						
Continues	0.73 (0.65–0.82)	<0.001	0.76 (0.67–0.85)	<0.001	0.80 (0.70–0.91)	0.001
Low	Ref.		Ref.		Ref.	
High	0.47 (0.32–0.68)	<0.001	0.52 (0.35–0.77)	<0.001	0.64 (0.41–1.00)	0.048
**END**						
Continues	0.82 (0.71–0.93)	0.003	0.82 (0.71–0.94)	0.006	0.82 (0.71–0.95)	0.009
Low	Ref.		Ref.		Ref.	
High	0.62 (0.38–1.00)	0.053	0.64 (0.39–1.03)	0.070	0.65 (0.39–1.07)	0.096

ASPECTS, the Alberta Stroke Program Early Computed Tomography Score; CALLY, C-reactive protein-albumin-lymphocyte; CI, confidence interval; END, early neurological deterioration; EVT, endovascular thrombectomy; Glu, fasting blood glucose; mRS, modified Rankin scale; NIHSS, National Institute of Health Stroke Scale; OR, odds ratio; WBC, white blood cell.

Model 1: was not adjusted for any covariates.

Model 2: was adjusted for medical history: smoke, hypertension, diabetes mellitus, hyperlipidemia, coronary heart disease, atrial fibrillation and cancer.

Model 3: was adjusted for variables with *p* < 0.1 in the univariable analysis after the back-ward selection: age, diabetes mellitus, WBC, Glu, successful recanalization, puncture to reperfusion time, baseline NIHSS, ASPECTS and mRS scores.

**Table 3:** Reclassification indexes for the CALLY index in different model.

	NRI (continuous)		NRI (categorical)		IDI	
Outcome	Value (95% CI)	*P* value	Value (95% CI)	*P* value	Value (95% CI)	*P* value
**Poor outcome**						
Model 2	0.214 (0.094–0.379)	<0.001	0.226 (−0.032 to 0.495)	0.148	0.041 (0.023–0.058)	<0.001
Model 3	0.160 (0.073–0.296)	0.004	0.053 (−0.032 to 0.296)	0.566	0.023 (0.011–0.036)	<0.001
**END**						
Model 2	0.123 (−0.001 to 0.386)	0.232	0.010 (−0.040 to 0.397)	0.936	0.019 (0.006–0.031)	0.003
Model 3	0.145 (−0.027 to 0.325)	0.124	0.001 (−0.067 to 0.268)	0.952	0.014 (0.002–0.026)	0.022

ASPECTS, the Alberta Stroke Program Early Computed Tomography Score; CALLY, C-reactive protein-albumin-lymphocyte; CI, confidence interval; END, early neurological deterioration; EVT, endovascular thrombectomy; IDI, Integrated Discrimination Improvement; Glu, fasting blood glucose; mRS, modified Rankin scale; NIHSS, National Institute of Health Stroke Scale; NRI, Net Reclassification Index; OR, odds ratio; WBC, white blood cell.

Model 2: was adjusted for smoke, hypertension, diabetes mellitus, hyperlipidemia, coronary heart disease, atrial fibrillation and cancer.

Model 3: was additionally adjusted for age, diabetes mellitus, WBC, Glu, successful recanalization, puncture to reperfusion time, baseline NIHSS, ASPECTS and mRS scores.

## Discussion

In this study, we identified a significant association between the CALLY index and the prognosis of AIS patients treated with EVT. We found that the CALLY index was inversely associated with the risk of poor functional outcome and END. The CALLY index may modestly enhance the predictive ability for poor functional outcomes and potentially offer superior prognostic value compared to classical inflammatory factors.

Previous studies with AIS patients treated with EVT have shown that inflammatory markers were significantly associated with poor outcomes. Study from the St George’s Hospital found that the combination of inflammatory marker was important predictor of 3-month clinical outcome after EVT. Higher NLR and lower lymphocyte-monocyte ratio were significantly associated with worse clinical outcome in AIS patients (Lux et al., 2020). Shin et al. computed various inflammation-based scores using laboratory data and demonstrated that the ratios of neutrophils, lymphocytes, monocytes, and high-density lipoprotein cholesterol all served as significant predictors of adverse outcomes (Oh et al., 2020). Pikija et al. reported the utility of the NLR as an independent predictor for the occurrence of intracerebral hemorrhage, identifying a cutoff value of 3.89 (Pikija et al., 2018). Finck et al. revealed that poor clinical outcomes and mortality were significantly more common when initial CRP levels were elevated (Finck et al., 2023). Our study yielded similar findings, demonstrating that inflammation plays a crucial role in the prognosis of EVT. Besides, we revealed that the integration of albumin can aid in identifying high-risk patients.

Unlike CRP-to-Albumin Ratio and Prognostic Nutritional Index, which selectively included CRP, albumin, and lymphocyte counts in their formulas, the CALLY index comprehensively integrated the inflammatory and nutritional markers and had a better performance in predicting prognosis than traditional markers (Iida et al., 2022). Albumin is regarded as a downregulated acute-phase protein, with levels typically decreasing during inflammatory states (Eckart et al., 2020). Beyond its crucial role in maintaining oncotic pressure, albumin exhibits anti-inflammatory properties, primarily through its antioxidant and antiplatelet activities (Taverna et al., 2013). Previous studies had shown that albumin levels were related to the risk of early cardiovascular events and mortality in patients with ischemic stroke in the federated research network (Bucci et al., 2024). Furthermore, researchers have exploratorily conducted studies on whether supplementing albumin can improve the prognosis of stroke patients. In rodent models of ischemic stroke, albumin therapy has been shown to exhibit significant neuroprotective effects, including reduced brain infarct volume, improved neurological scores, and normalization of diffusion-weighted imaging changes (Belayev et al., 1997; Tang et al., 2009). However, the ALIAS trial’s findings indicated that high-dose albumin in AIS patients failed to improve outcomes, possibly due to complications like pulmonary edema. Albumin should be regarded more as a prognostic indicator. The efficacy of albumin therapy required further clinical validation (Martin et al., 2016). Therefore, incorporating albumin into the calculation formula may enhance the predictive power of the CALLY index for patient prognosis compared to an index that only includes inflammatory markers.

The mechanism between the CALLY index and the prognosis of the EVT may be explained as follows. Firstly, CRP can disrupt endothelial progenitor cells and fibrinolysis, increase collagen destruction in monocytes, and promote the transformation of low-density lipoprotein cholesterol into foam cells via macrophage uptake (Bisoendial et al., 2010). Secondly, lymphocytes may negatively contribute to the pathogenesis of brain injury. Interrupting the entry of lymphocytes into infarcted brain tissues can reduce the severity of the injury, indicating that, similar to neutrophils, lymphocytes play an overall protective role (Becker et al., 2001). Thirdly, albumin demonstrates anti-atherogenic effects through the inhibition of low-density lipoprotein cholesterol oxidation, suppression of platelet activation and aggregation, and enhancement of prostacyclin bioavailability (Purdon and Rao, 1989). Moreover, components of the CALLY index are also involved in the pathophysiology of ischemia–reperfusion injury after stroke. Ischemia–reperfusion injury is a complex cascade involving interactions among vascular endothelium, interstitial spaces, circulating cells, and numerous biochemical entities (Zhang et al., 2022). Inflammation is considered a key mediator of ischemia–reperfusion injury, with substantial evidence highlighting the role of innate immunity (Li et al., 2021). CRP is a component of the nonspecific acute-phase response to inflammation and tissue damage following ischemia–reperfusion. The proposed mechanism of CRP’s involvement is through local activation of the complement system, as evidenced by its deposition on myocardial cells within the infarcted area (den Hertog et al., 2009; Shen et al., 2024). The activation of lymphocytes in macrophage recruitment and the enhancement of vascular endothelial growth factor may mediate collateral vessel development after ischemia–reperfusion injury (Linfert et al., 2009). Nutritional status also influences the progression of vascular injury after ischemic events (Cai et al., 2022; Hu et al., 2024). Microvascular flow and functional capillary density were maintained in animals treated with albumin, suggesting that albumin preserved vascular permeability, as evidenced by the extravasation of fluorescently labeled dextran (Belcher et al., 2022).

We found that the CALLY index exhibited a moderate discriminative ability (AUC = 0.628) and a decreasing trend in association with the risk of poor functional outcome after EVT, which was comparable to previous studies on survival rates in colorectal cancers (AUC = 0.666) (Yang et al., 2023). Based on the optimal cut-off value for the CALLY index [10^ (−0.635)], patients with a CALLY index below this threshold may experience an increased risk due to the inflammation reaction, poor nutritional status, and impaired immune function, suggesting the necessity for anti-inflammatory and nutritional interventions (Luo et al., 2024). In contrast, patients with a CALLY index above the threshold may be more stable and are more likely to have a favorable outcome. The threshold of the CALLY index may offer valuable clinical insights, serving as a tool for risk stratification, guiding targeted interventions, and personalized patient care.

Our study suggested that the CALLY index’s influence may be more significant in elderly patients and those with atrial fibrillation. Previous research indicated that older patients were at a higher risk for inflammatory diseases and sarcopenia (Li et al., 2024). Therefore, evaluating the relationship between inflammatory and nutritional states and clinical outcomes using the CALLY index may be critical in the elderly population. Patients with atrial fibrillation typically require oral anticoagulation. The administration of anticoagulant therapy was reported to modulate inflammatory factors via protease-activated receptor signaling and to mediate anti-inflammatory effects in critically ill patients (Jannati et al., 2024). Anticoagulant therapy may theoretically reduce the inflammatory burden, increase the CALLY index, and thereby improve outcomes after EVT, which required further investigation in clinical practice.

To the best of our knowledge, this was the first study to investigate the association between the CALLY index and AIS patients treated with EVT. However, there were several limitations in this study. Firstly, this study was a retrospective analysis with a relatively small sample size. The results should be interpreted with caution like subgroup analyses. Further validation of our findings in large-scale prospective studies was strongly recommended. Secondly, our investigation only collected CRP, lymphocyte count, and albumin levels at admission and did not analyze their dynamic changes or their impact on the prognosis of EVT. Thirdly, our study contained unmeasured confounders with inherent biases, such as anti-inflammatory medications, and we were unable to establish a causal relationship between the CALLY index and functional outcome. Fourthly, the study was conducted in the Chinese population, which may limit the generalizability of the findings, as genetic and environmental factors could influence the CALLY index and stroke care processes. Lastly, the efficacy of anti-CRP interventions in improving clinical outcomes for AIS patients was not evaluated. A prospective study evaluating the effect of anti-inflammatory or nutritional interventions in larger cohorts of AIS patients is warranted.

## Conclusion

In conclusion, our study findings suggested that a lower CALLY index was an independent predictor of poor functional outcome after EVT. The CALLY index could serve as a practical, cost-effective, and promising predictive biomarker for poor outcomes in AIS patients treated with EVT and facilitate triaging high-risk patients who may benefit from early interventions, such as adjuvant anti-inflammatory therapy or intensive nutritional support.

## Data Availability

The raw data supporting the conclusions of this article will be made available by the authors, without undue reservation.
