# Supplementary material for: Lower CALLY index levels indicate higher poor functional outcome risk in acute ischemic stroke patients treated with endovascular thrombectomy
Source: Front Aging Neurosci. 2025 Apr 25;17:1587861. doi: 10.3389/fnagi.2025.1587861 (PMC12061938; doi:10.3389/fnagi.2025.1587861)
Supplement: Supplementary file 1 [file Data_Sheet_1.PDF]

Supplementary materials

Supplementary Figure 1. The distribution of CALLY index before and after log transformation.

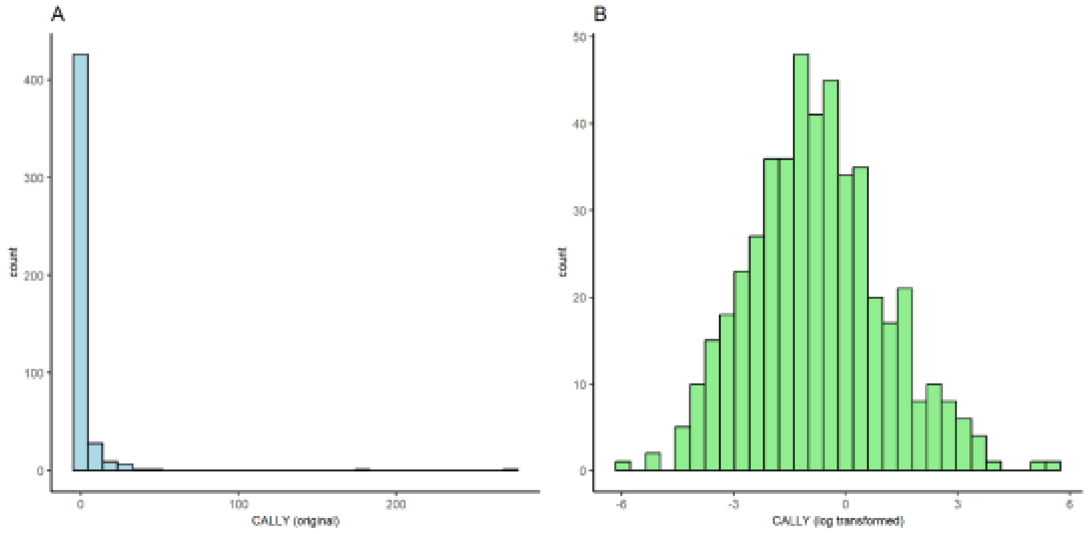

Abbreviations: The distribution of the CALLY index suggested skewness, which was corrected by a logarithmic transformation, resulting in a normal distribution. CALLY, CRP-Albumin-Lymphocyte.

Supplementary Figure 2. The distribution of log-transformed CALLY index in the histogram and Q-Q plots.

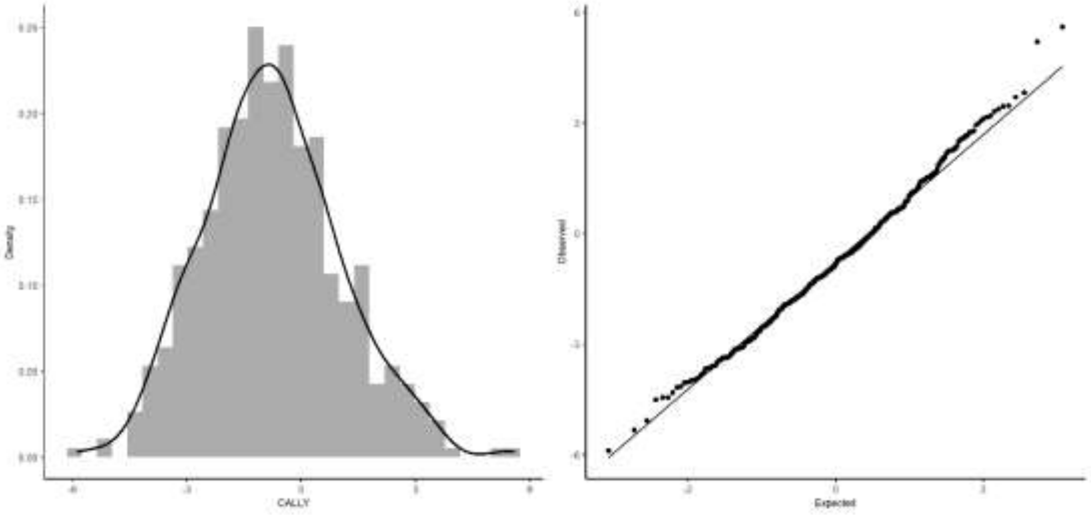

Abbreviations: CALLY, CRP-Albumin-Lymphocyte.

Supplementary Figure 3. Flow diagram of the study.

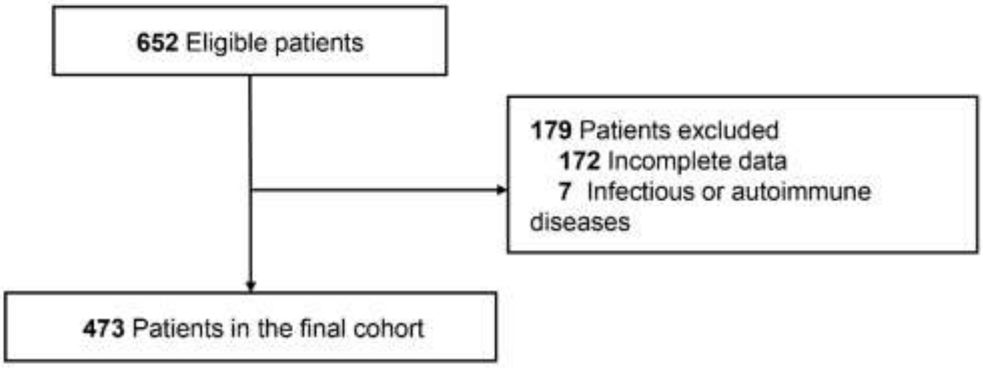

Supplementary Figure 4. ROC curve for inflammatory markers.

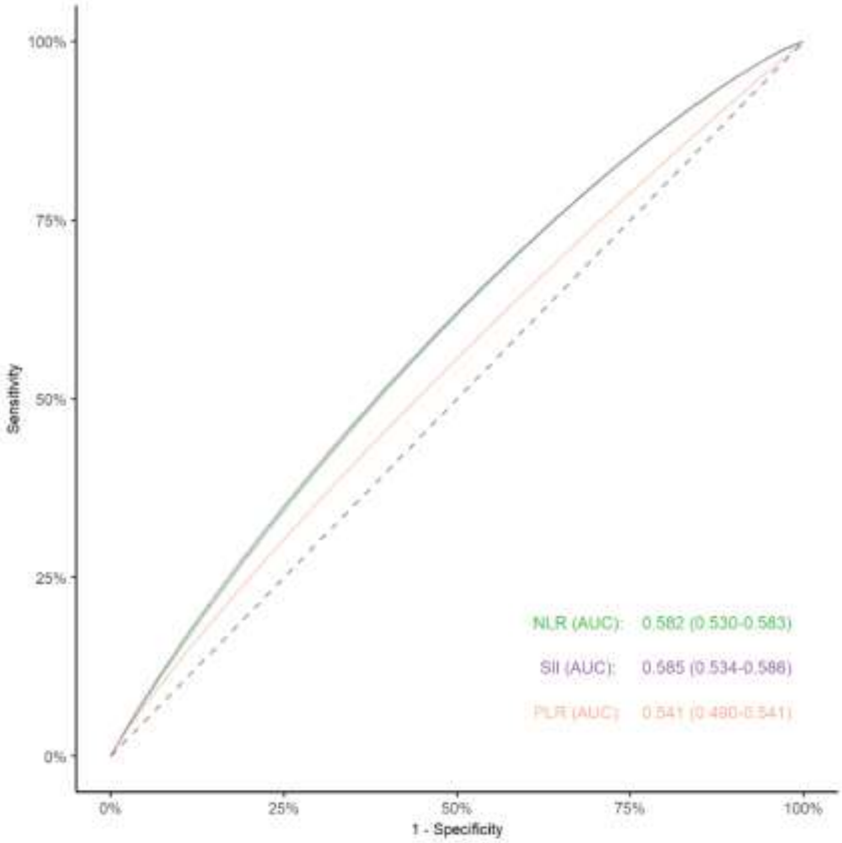

Abbreviations: AUC, area under the curve; EVT, endovascular thrombectomy; NLR, systemic immunoinflammatory index; PLR, PLR, platelet / lymphocyte; ROC, receiver operating characteristic curve; SII, systemic immunoinflammatory index. The Delong's test showed that the CALLY index was better than NLR (*P* = 0.072), SII (*P* = 0.099) and PLR (*P* = 0.002).

Supplementary Table 1. Characteristics of the study population according to the study centers.

Variable	Xishan People's Hospital of Wuxi City (n = 285)	The Affiliated Zhangjiagang Hospital of Soochow University (n = 188)	*P* value
Age (years)	71.0 [63.0, 79.0]	72.0 [65.0, 80.0]	0.315
Gender / Male, n (%)	192 (67.4)	110 (58.5)	0.062
Physical examination			
Height (cm)	166.0 [160.0, 170.0]	165.0 [160.0, 170.0]	0.463
Weight (kg)	65.0 [60.0, 75.0]	66.2 [57.0, 75.0]	0.832
SBP (mmHg)	139.3 (22.5)	139.0 (21.7)	0.876
DBP (mmHg)	83.5 (12.9)	84.6 (14.1)	0.372
Comorbidities, n (%)			
Smoking	120 (42.1)	63 (33.5)	0.075
Drinking	73 (25.6)	36 (19.1)	0.128
Hypertension	204 (71.6)	134 (71.3)	1.000
Diabetes mellitus	76 (26.7)	55 (29.3)	0.61
Hyperlipidemia	31 (10.9)	14 (7.4)	0.278
Coronary artery disease	37 (13.0)	35 (18.6)	0.124
Atrial fibrillation	70 (24.6)	59 (31.4)	0.127
Cancer	8 (2.8)	8 (4.3)	0.553
Laboratory values, (median [IQR])			
UA (μmol/L)	320.0 [245.0, 393.9]	300.0 [251.2, 360.0]	0.130
Serum creatinine (μmol/L)	72.0 [61.0, 90.0]	71.4 [58.0, 86.0]	0.426
CRP (mg/L)	8.1 [3.0, 27.0]	10.1 [4.0, 27.0]	0.354
Albumin (g/L)	39.5 [35.8, 42.4]	38.3 [35.1, 41.6]	0.120
WBC count ($\times 10^9$/L)	9.6 [7.3, 12.2]	8.9 [7.1, 12.3]	0.294
Neutrophil count ($\times 10^9$/L)	8.0 [5.7, 10.4]	7.2 [5.4, 10.4]	0.254
Lymphocyte count ($\times 10^9$/L)	1.1 [0.8, 1.5]	1.0 [0.7, 1.5]	0.390
Glu (mmol/L)	6.6 [5.6, 8.6]	6.8 [5.8, 8.8]	0.588
PLT count ($\times 10^9$/L)	172.0 [138.0, 216.0]	171.0 [134.5, 218.0]	0.632
TOAST, n (%)			0.341
CES	120 (42.1)	91 (48.4)	
LAA	139 (48.8)	79 (42.0)	
Other	26 (9.1)	18 (9.6)	
IVT, n (%)	123 (43.2)	56 (29.8)	0.005
Angiographic outcomes			
Number of attempts (n)	1.0 [1.0, 2.0]	1.0 [1.0, 2.0]	0.105
mTICI 2b / 3, n (%)	262 (91.9)	180 (95.7)	0.147
OTP (min)	285.0 [180.0, 444.0]	315.0 [193.8, 631.2]	0.015
PTR (min)	58.0 [40.0, 81.0]	65.0 [47.8, 105.0]	0.003
ASITN/SIR 2-3, n (%)	40 (14.0)	24 (12.8)	0.797

Clinical scores (points)			
Baseline NIHSS	13.0 [10.0, 16.0]	14.0 [10.0, 18.0]	0.020
Baseline mRS	0.0 [0.0, 0.0]	0.0 [0.0, 0.0]	0.024
Baseline ASPECTS	9.0 [8.0, 9.0]	9.0 [8.0, 9.0]	0.176
Occlusion site, n (%)			0.056
ICA	61 (21.4)	49 (26.1)	
MCA-M1	157 (55.1)	86 (45.7)	
MCA-M2	17 (6.0)	22 (11.7)	
T occlusion	50 (17.5)	31 (16.5)	
END, n (%)	49 (17.2)	39 (20.7)	0.395
Poor outcome	125 (43.9)	89 (47.3)	0.516

Abbreviations: ASITN/SIR, the American Society of Interventional and Therapeutic Neuroradiology/Society of Interventional Radiology; ASPECTS, the Alberta Stroke Program Early Computed Tomography Score; CALLY, C-reactive protein-albumin-lymphocyte; CES, cardioembolism; CRP, C-reactive protein; DBP, diastolic blood pressure; END, early neurological deterioration; EVT, endovascular thrombectomy; Glu, fasting blood glucose; ICA, internal carotid artery; ICH, intracranial hemorrhage; IVT, intravenous thrombolysis; LAA, large artery atherosclerosis; MCA, middle cerebral artery; mRS, modified Rankin Scale Score; mTICI, modified Thrombolysis in Cerebral Infarction Score; NIHSS, National Institute of Health Stroke Scale; OTP, from onset to puncture; PTR, from puncture to recanalization; SBP, systolic blood pressure; TOAST, the trial of ORG 10172 in Acute Stroke Treatment classification; UA, uric acid; WBC, white blood cell.

Supplementary Table 2. Characteristics of the study population according to the CALLY index threshold grouping.

Variable	Low CALLY (n = 257)	High CALLY (n = 216)	*P* value
Age (years)	73.0 [65.0, 80.0]	69.0 [60.0, 77.2]	0.004
Gender / Male, n (%)	164 (63.8)	138 (63.9)	1.000
Physical examination			
Height (cm)	139.1 (22.7)	139.3 (21.6)	0.897
Weight (kg)	84.3 (13.7)	83.5 (13.0)	0.542
SBP (mmHg)	165.0 [160.0, 170.0]	165.0 [160.0, 170.0]	0.367
DBP (mmHg)	65.0 [57.8, 75.0]	69.5 [60.0, 75.0]	0.203
Comorbidities, n (%)			
Smoking	98 (38.1)	85 (39.4)	0.860
Drinking	55 (21.4)	54 (25.0)	0.414
Hypertension	189 (73.5)	149 (69.0)	0.321
Diabetes mellitus	75 (29.2)	56 (25.9)	0.493
Hyperlipidemia	24 (9.3)	21 (9.7)	1.000
Coronary artery disease	44 (17.1)	28 (13.0)	0.260
Atrial fibrillation	76 (29.6)	53 (24.5)	0.262
Cancer	12 (4.7)	4 (1.9)	0.152
Laboratory values, (median [IQR])			
UA (μmol/L)	311.6 [250.0, 390.7]	310.5 [246.4, 382.2]	0.794
Serum creatinine (μmol/L)	75.0 [60.2, 95.0]	70.8 [58.3, 83.5]	0.030
CRP (mg/L)	26.5 [11.3, 60.8]	2.7 [1.2, 5.0]	<0.001
Albumin (g/L)	37.8 [33.7, 41.0]	40.0 [37.0, 43.2]	<0.001
WBC count ($\times10^9$/L)	9.3 [7.2, 12.7]	9.2 [7.1, 11.8]	0.371
Neutrophil count ($\times10^9$/L)	8.0 [5.8, 11.1]	7.3 [5.3, 9.7]	0.040
Lymphocyte count ($\times10^9$/L)	0.9 [0.6, 1.3]	1.3 [0.9, 1.7]	<0.001
Glu (mmol/L)	6.8 [5.7, 8.8]	6.6 [5.7, 8.0]	0.527
PLT count ($\times10^9$/L)	168.0 [130.0, 218.0]	178.5 [149.8, 216.0]	0.081
TOAST, n (%)			0.804
CES	118 (45.9)	93 (43.1)	
LAA	115 (44.7)	103 (47.7)	
Other	24 (9.3)	20 (9.3)	
IVT, n (%)	96 (37.4)	83 (38.4)	0.885
Angiographic outcomes			
Number of attempts (n)	1.0 [1.0, 2.0]	1.0 [1.0, 2.0]	0.055
mTICI 2b / 3, n (%)	239 (93.0)	203 (94.0)	0.807
OTP (min)	315.0 [200.0, 500.0]	260.0 [173.8, 491.2]	0.047
PTR (min)	63.0 [45.0, 97.0]	58.5 [40.0, 84.2]	0.016
ASITN/SIR 2-3, n (%)	35 (13.6)	29 (13.4)	1.000
Clinical scores (points)			
Baseline NIHSS	14.0 [10.0, 18.0]	12.0 [9.0, 16.0]	0.004
Baseline mRS	0.0 [0.0, 0.0]	0.0 [0.0, 0.0]	0.127

Baseline ASPECTS	9.0 [8.0, 9.0]	9.0 [8.0, 9.0]	0.308
Occlusion site, n (%)			0.566
ICA	66 (25.7)	44 (20.4)	
MCA-M1	129 (50.2)	114 (52.8)	
MCA-M2	21 (8.2)	18 (8.3)	
T occlusion	41 (16.0)	40 (18.5)	
END, n (%)	56 (21.8)	32 (14.8)	0.068
Poor outcome	138 (53.7)	76 (35.2)	<0.001

Abbreviations: ASITN/SIR, the American Society of Interventional and Therapeutic Neuroradiology/Society of Interventional Radiology; ASPECTS, the Alberta Stroke Program Early Computed Tomography Score; CALLY, C-reactive protein-albumin-lymphocyte; CES, cardioembolism; CRP, C-reactive protein; DBP, diastolic blood pressure; END, early neurological deterioration; EVT, endovascular thrombectomy; Glu, fasting blood glucose; ICA, internal carotid artery; ICH, intracranial hemorrhage; IVT, intravenous thrombolysis; LAA, large artery atherosclerosis; MCA, middle cerebral artery; mRS, modified Rankin Scale Score; mTICI, modified Thrombolysis in Cerebral Infarction Score; NIHSS, National Institute of Health Stroke Scale; OTP, from onset to puncture; PTR, from puncture to recanalization; SBP, systolic blood pressure; TOAST, the trial of ORG 10172 in Acute Stroke Treatment classification; UA, uric acid; WBC, white blood cell.

Supplementary Table 3. The relationship between other inflammation indexes and outcomes after EVT.

	Model 1		Model 2		Model 3	
**Outcome**	**OR (95% CI)**	***P* value**	**OR (95% CI)**	***P* value**	**OR (95% CI)**	***P* value**
**Poor outcome**						
Log (NLR)	1.43 (1.15-1.79)	0.002	1.46 (1.15-1.87)	0.002	1.18 (0.87-1.60)	0.297
Log (SII)	1.41 (1.16-1.73)	0.001	1.47 (1.18-1.84)	0.001	1.32 (1.01-1.75)	0.051
Log (PLR)	1.30 (1.01-1.69)	0.046	1.32 (1.01-1.79)	0.055	1.37 (1.01-1.92)	0.056
**END**						
Log (NLR)	1.13 (0.86-1.49)	0.395	1.11 (0.84-1.49)	0.460	1.09 (0.78-1.53)	0.607
Log (SII)	1.02 (0.81-1.29)	0.878	1.00 (0.79-1.27)	0.980	1.00 (0.76-1.33)	0.986
Log (PLR)	0.95 (0.71-1.29)	0.715	0.92 (0.69-1.26)	0.582	0.95 (0.70-1.32)	0.770

Abbreviations: ASPECTS, the Alberta Stroke Program Early Computed Tomography Score; CI, confidence interval; END, early neurological deterioration; EVT, endovascular thrombectomy; Glu, fasting blood glucose; mRS, modified Rankin scale; NIHSS, National Institute of Health Stroke Scale; NLR, neutrophil / lymphocyte; OR, odds ratio; PLR, platelet / lymphocyte; SII, systemic immunoinflammatory index; WBC, white blood cell.

Model 1: was not adjusted for any covariates.

Model 2: was adjusted for smoke, hypertension, diabetes mellitus, hyperlipidemia, coronary heart disease, atrial fibrillation and cancer.

Model 3: was adjusted for age, diabetes mellitus, WBC, Glu, successful recanalization, puncture to reperfusion time, baseline NIHSS, ASPECTS and mRS scores.

Supplementary Table 4. The relationship between post-EVT CALLY index and outcomes after EVT.

Outcome	Model 1 OR (95% CI)	*P* value	Model 2 OR (95% CI)	*P* value	Model 3 OR (95% CI)	*P* value
**Poor outcome**						
Decreased CALLY index	Ref		Ref		Ref	
Increased CALLY index	0.57 (0.35-0.94)	0.027	0.50 (0.29-0.85)	0.011	0.38 (0.20-0.70)	0.002
**END**						
Decreased CALLY index	Ref		Ref		Ref	
Increased CALLY index	1.04 (0.56-1.94)	0.896	0.96 (0.51-1.83)	0.900	0.99 (0.51-1.92)	0.971

Abbreviations: ASPECTS, the Alberta Stroke Program Early Computed Tomography Score; CI, confidence interval; END, early neurological deterioration; EVT, endovascular thrombectomy; Glu, fasting blood glucose; mRS, modified Rankin scale; NIHSS, National Institute of Health Stroke Scale; NLR, neutrophil / lymphocyte; OR, odds ratio; PLR, platelet / lymphocyte; SII, systemic immunoinflammatory index; WBC, white blood cell.

Model 1: was not adjusted for any covariates.

Model 2: was adjusted for smoke, hypertension, diabetes mellitus, hyperlipidemia, coronary heart disease, atrial fibrillation and cancer.

Model 3: was adjusted for age, diabetes mellitus, WBC, Glu, successful recanalization, puncture to reperfusion time, baseline NIHSS, ASPECTS and mRS scores.
